# Conversion of crude oil to methane by a microbial consortium enriched from oil reservoir production waters

**DOI:** 10.3389/fmicb.2014.00197

**Published:** 2014-05-05

**Authors:** Carolina Berdugo-Clavijo, Lisa M. Gieg

**Affiliations:** Petroleum Microbiology Research Group, Department of Biological Sciences, University of CalgaryCalgary, AB, Canada

**Keywords:** crude oil, hydrocarbon methanogenesis, crude oil reservoir, pyrotag sequencing, alkylsuccinates

## Abstract

The methanogenic biodegradation of crude oil is an important process occurring in petroleum reservoirs and other oil-containing environments such as contaminated aquifers. In this process, syntrophic bacteria degrade hydrocarbon substrates to products such as acetate, and/or H_2_ and CO_2_ that are then used by methanogens to produce methane in a thermodynamically dependent manner. We enriched a methanogenic crude oil-degrading consortium from production waters sampled from a low temperature heavy oil reservoir. Alkylsuccinates indicative of fumarate addition to C_5_ and C_6_ *n*-alkanes were identified in the culture (above levels found in controls), corresponding to the detection of an alkyl succinate synthase encoding gene (*assA/masA*) in the culture. In addition, the enrichment culture was tested for its ability to produce methane from residual oil in a sandstone-packed column system simulating a mature field. Methane production rates of up to 5.8 μmol CH_4_/g of oil/day were measured in the column system. Amounts of produced methane were in relatively good agreement with hydrocarbon loss showing depletion of more than 50% of saturate and aromatic hydrocarbons. Microbial community analysis revealed that the enrichment culture was dominated by members of the genus *Smithella, Methanosaeta*, and *Methanoculleus*. However, a shift in microbial community occurred following incubation of the enrichment in the sandstone columns. Here, *Methanobacterium* sp. were most abundant, as were bacterial members of the genus *Pseudomonas* and other known biofilm forming organisms. Our findings show that microorganisms enriched from petroleum reservoir waters can bioconvert crude oil components to methane both planktonically and in sandstone-packed columns as test systems. Further, the results suggest that different organisms may contribute to oil biodegradation within different phases (e.g., planktonic vs. sessile) within a subsurface crude oil reservoir.

## Introduction

In hydrocarbon-impacted subsurface environments, fuel components can be anaerobically biodegraded via a number of anaerobic electron accepting processes including nitrate, iron, and sulfate reduction (Widdel et al., 2010). However, when available electron acceptors are depleted in such environments, hydrocarbon biodegradation has to proceed via methanogenesis. Methanogenic hydrocarbon metabolism involves the interaction between syntrophic bacteria and methanogens. Although methanogenic oil biodegradation is considered a low energy yielding process (Schink, 1997), it is thermodynamically feasible when intermediate products generated by syntrophic bacteria are kept at low concentrations by methanogens (Dolfing et al., 2007). The biodegradation of hydrocarbons under methanogenic conditions has been widely investigated for a variety of crude oil components such as *n*-alkanes (Zengler et al., 1999; Anderson and Lovley, 2000), benzene (Grbic'-Galic' and Vogel, 1987; Ulrich and Edwards, 2003), toluene (Grbic'-Galic' and Vogel, 1987; Godsy et al., 1992; Edwards and Grbic'-Galic', 1994), and polycyclic aromatic hydrocarbons (Chang et al., 2006; Berdugo-Clavijo et al., 2012; Zhang et al., 2012b). Only more recently have reports emerged demonstrating the susceptibility of whole crude oil to methanogenic biodegradation (Townsend et al., 2003; Jones et al., 2008; Gieg et al., 2010) and work is ongoing to elucidate the mechanism(s) involved in the initial activation of hydrocarbons under these conditions. Addition to fumarate as an initial hydrocarbon activation mechanism under anoxic conditions was initially demonstrated with toluene under nitrate-reducing conditions (Biegert et al., 1996) and subsequently for other alkyl-substituted monoaromatic compounds (reviewed in Foght, 2008; Widdel et al., 2010). Likewise, *n*-alkanes were shown to be activated via addition to fumarate by nitrate- and sulfate-reducing bacteria (e.g., Kropp et al., 2000; Rabus et al., 2001; Callaghan et al., 2006). Fumarate addition has been shown in methanogenic toluene-degrading enrichments by quantification, expression, or detection of benzylsuccinate synthase genes (Beller et al., 2002; Washer and Edwards, 2007; Sun et al., 2014), as well as by metabolite analysis (Fowler et al., 2012). Although fumarate addition genes (e.g., *ass/mas* for alkanes or *bss* for toluene) have been identified in methanogenic oil-degrading enrichments (Zhou et al., 2012; Aitken et al., 2013; Tan et al., 2013) and samples from oil-contaminated environments (Callaghan et al., 2010), it is still uncertain whether this metabolic pathway occurs during methanogenic oil biodegradation. Other putative activation mechanisms may include carboxylation, hydroxylation, or methylation, all of which have been reported to occur under other electron-accepting conditions (e.g., reviewed in Foght, 2008; Widdel et al., 2010).

The understanding of methanogenic crude oil biodegradation can contribute to a number of biotechnological applications related to bioremediation (Kazy et al., 2010; Callaghan, 2013) and enhanced oil or energy recovery from marginal oil reservoirs (Parkes, 1999; Gieg et al., 2008; Jones et al., 2008). For the latter application, it is feasible that entrained oil can be bioconverted to methane that can be recovered as an energy source or that can be used to re-pressurize the reservoir and reduce oil viscosity via stimulating indigenous subsurface microbial communities or via bioaugmentation (Gieg et al., 2008; Gray et al., 2009, 2010). Overall, a better understanding of the metabolic processes and key microorganisms involved in converting crude oil to methane is still necessary to assess the feasibility and challenges of this technology (Gray et al., 2010).

In this study, we established a methanogenic crude oil-degrading consortium from production waters of a low temperature heavy oil reservoir, identified some putative hydrocarbon metabolites, and characterized the microbial community using pyrotag sequencing. In addition, we assessed whether the syntrophic enrichment could bioconvert crude oil components to methane in sandstone-packed, residual oil-containing columns in order to more closely simulate a mature field and estimate hydrocarbon consumption, determine rates of methanogenesis, and identify key microorganisms that may be contributing to hydrocarbon methanogenesis in crude oil reservoirs.

## Materials and methods

### Development of a crude oil-degrading enrichment culture

A methanogenic enrichment culture was initially obtained from a mixture of production waters of a low temperature reservoir wherein nitrate is being assessed to treat souring (Agrawal et al., 2012). The production waters were initially amended with 0.5–1 mM of phosphate and 5% (by volume) crude oil. Following the detection of methane, a secondary enrichment culture was developed by transferring 20 mL of the original culture into 20 mL of a bicarbonate-buffered (pH 7.1), anoxic minimal medium (headspace contained N_2_/CO_2_, 90/10 by vol) that contained resazurin and was reduced with cysteine sulfide (McInerney et al., 1979). The enrichment was amended with 0.5 mL of light crude oil that was preflushed with N_2_; substantial methane was produced from this secondary enrichment (unpublished data). To establish the experiments for this study, the microbial culture was again transferred (10% by volume), in triplicate, into sterile anoxic medium (50 mL, described above) amended with 0.5 mL of light crude oil (°API = 37) or 0.2 mL of heavy crude oil (°API = 16). In addition, inoculated control incubations without crude oil were prepared in parallel to account for any background production of methane. Enrichments were incubated in the dark at 33°C for approximately 28 weeks.

### Chemical analyses

Methane production from the oil-degrading enrichments and columns was monitored over time by injecting 0.2 mL of a serum bottle head space into a HP model 5890 gas chromatograph (GC) equipped with a flame ionization detector (FID) as previously described (Berdugo-Clavijo et al., 2012). Carbon dioxide was also monitored from the column experiments using HP-MS and HP-Plot/Q capillary columns (30 m × 0.30 mm × 0.25 μm) installed into a GC equipped with a thermal conductivity detector (GC-TCD, Agilent Technologies). Headspace samples (0.1 mL) were analyzed in a split mode (2:1) via an injector held at 250°C. The detector and oven temperatures were set at 200 and 80°C, respectively.

Supernatants (40 mL) from the crude-oil degrading enrichments and controls were subsampled when substantial amounts of methane were produced. Samples were acidified with HCl (to pH = 2), extracted with three volumes of ethyl acetate that were dried over anhydrous sodium sulfate, and were initially concentrated by rotary evaporation at 60°C. Samples were further concentrated under a stream of N_2_ to a volume of 100 μL then silylated with 100 μL of *N*, *O*-bis-(trimethylsilyl) trifluoroacetamide (Thermo Scientific, Waltham, MA) for 20 min at 60°C. All samples were prepared to the exact same volume. Sample components were separated and identified using an Agilent 7890A GC equipped with an HP-5MS column (50 m × 0.25 mm × 0.25 μm; Agilent) and an Agilent 5975C mass selective detector. The oven temperature was held at 45°C for 5 min, then increased at a rate of 4°C/min to 270°C, then held at this temperature for 5 min. The injector, operated in split mode (50:1) was held at 270°C. Putative hydrocarbon metabolites were identified through mass spectral analysis and comparisons to literature reports, or confirmed by matching GC retention times and MS profiles with commercially available authentic standards.

### DNA analysis

In order to characterize the microbial community of the crude-oil degrading enrichment, genomic DNA was isolated. A liquid sample (5 mL) withdrawn from the culture was centrifuged at 14,000 rpm for 5 min to pellet the cells, then DNA was extracted using a commercially available kit (FastDNA Spin Kit for Soil; MP Biomedicals). The isolated DNA was amplified using universal primers 926F (AAA CTY AAA KGA Att GAC GG) and 1392R (ACG GGC GGT GTG TRC) with PCR Master Mix in a 50-μ L reaction (Taq DNA polymerase reagents (500U); Qiagen, Mississauga, Canada) in a two-round PCR method. The conditions for the primary PCR were as follows: 95°C, 3 min; 25 cycles of 95°C, 30 s; 55°C, 45 s; 72°C, 90 s; 72°C, 10 min; final hold at 4°C. The secondary PCR was done with FLX Titanium amplicon primers 454T-RA and 454T-FB, which contained an adaptor and barcode at the 5′ end adjacent to the 926F and 1392R primers. Primer 454T-RA has a 25 nucleotide A-adaptor (CGTATCGCCTCCCTCGCGCCATCAG), whereas primer 454T-FB has a 25 nucleotide B-adaptor sequence (CTATGCGCCTTGCCAGCCCGCTCAG). The conditions of the second round PCR were as follow: 95°C for 3 min, 10 cycles of 95°C for 30 s, 55°C for 45 s, 72°C for 90 s and a final extension step at 72°C for 10 min. PCR products were purified with a QIAquick PCR Purification Kit (Qiagen, Mississauga, Canada) and quantified with a Qubit Fluorometer (Invitrogen, Carlsbad, USA). PCR products (200 ng) were sent to the McGill University and Genome Quebec Innovation Centre where they were analyzed by pyrosequencing using a GS FLX Titanium Series Kit XLR70 (Roche Diagnostics Corporation). Data analysis was conducted using Phoenix 2, an in-house ssu rRNA pipeline data system that incorporates a series of stringent tests to remove low quality reads and reduce sequencing errors (Soh et al., 2013). For our analysis we used the taxonomic annotation results generated with the Silva database at a 5% cutoff clustering distance.

In addition, the alkyl- and benzylsuccinate synthase genes (*assA* and *bssA*) were assayed in the extracted DNA from the oil-degrading enrichment culture with nine established primer sets (Callaghan et al., 2010) using a 50 μ L reaction PCR Master mixture with Taq DNA polymerase reagent (500 U) (Qiagen, Mississauga, Canada) and the following PCR conditions: 95°C for 3 min, followed by 40 cycles of 95°C for 45 s, 55°C for 1 min, 72°C for 2 min, and a final extension step at 72°C for 10 min. PCR products were observed on a 1% agarose gel and detected by SYBR safe gel stain to visualize amplified gene single fragments of the expected sizes. All reactions were conducted in triplicate. Amplified bands were gel extracted and purified using the QIAquick gel extraction kit (Qiagen, Mississauga, Canada). Recovered samples were sequenced and queried against the ENA database (European Nucleotide Archive, http://www.ebi.ac.uk/ena/home).

### Establishment of residual oil simulating sandstone-packed columns

Following the development of the methanogenic enrichment culture, it was used as an inoculum to determine whether residual crude oil could be converted into methane in sandstone-packed column systems simulating mature oil fields (Figure S1). All preparation steps were carried out inside an anaerobic glove bag containing N_2_/CO_2_ (90/10) using components that were sterilized by autoclaving. Sterile glass columns (30 mL) fitted with a layer of polymeric mesh at the bottom were packed with 40 g of crushed Berea sandstone core (that was sieved to <250 μm grain size). The tops of the columns were sealed with rubber stoppers that were modified to incorporate an effluent port. Residual-oil conditions were simulated in the columns by first injecting an anoxic mineral salts medium (McInerney et al., 1979), then flooding the columns with light crude oil until saturation was obtained, and finally injecting anoxic medium again into the columns to displace the non-absorbed crude oil. A mineral salts medium was used to provide N, P, and other essential nutrients so that they were not limiting the hydrocarbon biodegradation processes; such biostimulation would likely be required for a field application to enhance the recovery of gas from oil in marginal reservoirs (Gray et al., 2010). All injections were done with Tygon tubing connected to the bottom of the column and using a multichannel peristaltic pump (Minipuls 3 Gilson) at a flow rate of 27 mL/h. Residual-oil simulating columns were ready when the water and oil volume ratio reached equilibrium. Between 40 and 50% of the crude oil initially injected remained trapped in the columns. The average porosity of the columns was 32% ± 5 (*n* = 6) and the estimated permeability was 1100 mD. Subsequently, columns were inoculated with 4 mL of the light oil-degrading enrichment culture by first injecting 2 mL of the inoculum followed by a 5 mL injection with sterile anoxic mineral medium (added at 5.3 mL/h) to push the microbial culture through the column, and then repeating the injection again with 2 mL inoculum and 3 mL of medium. These volumes were added to help ensure that the pore volumes throughout the column contained the inoculum. Triplicate oil-containing, inoculated columns were prepared. In addition, duplicate inoculated, oil-free control columns were prepared to account for any background methane while duplicate uninoculated control columns amended with crude oil were also prepared. Once inoculated, the residual-oil simulating columns were “shut-in” to allow for hydrocarbon methanogenesis to occur. The columns were incubated in the dark in an anoxic glove bag filled with N_2_/CO_2_ (90/10) at room temperature for up to 48 weeks. In addition, parallel columns inoculated with produced water from the same oilfield used for the establishment of the enrichment culture were prepared by flushing with sterile anoxic distilled water or anoxic medium to examine whether differences in methane production would be observed in the presence or absence of nutrients (such as N and P). However, the column set inoculated with a known hydrocarbon-degrading methanogenic enrichment culture prepared by flushing with bicarbonate-buffered anoxic minimal medium was the focus of this study.

Methane production from the columns was collected over time in argon-flushed serum bottles (25 mL) attached at the top of each column. These bottles were subsampled periodically to determine CH_4_ concentrations by GC-FID and CO_2_ concentrations by GC-TCD as described above. After 45 weeks of incubation (315 days), sand-packed columns that were actively producing methane over time relative to the oil-unamended controls were flushed with anoxic medium (approximately 10 mL representing the aqueous pore volume) to collect any byproducts potentially formed during crude oil degradation. Specifically, acetate was measured by ion chromatography as previously described (Grigoryan et al., 2008). Sand-packed incubations were then taken out of the anaerobic hood and aseptically opened for analysis. The sandstone material was mixed aseptically in an attempt to attain a homogenous mixture. Duplicate 500-mg samples were immediately removed for DNA extraction, PCR amplification, and pyrotag sequencing as described above. In addition, duplicate subsamples were removed for hydrocarbon analysis. Crude oil hydrocarbons entrained in the sandstone were recovered with a Soxhlet extractor method by running methylene chloride continuously through 10 g of sand for 8 h. About 0.8 g of crude oil was extracted, and a portion of the oil (0.2 g) was used for saturate and aromatic component separation. Extracted oil was passed through a silica column developed with 10 mL of 100% pentane, 20 mL of 20% methylene chloride in pentane, and 25 mL of 50% methylene chloride in pentane, to allow for the separation of saturate and aromatic fractions (Fedorak and Westlake, 1981). Squalene and *p*-terphenyl (0.48 μmol) were used as internal standards for the saturate and aromatic fractions, respectively. Extracted fractions were collected in separate tubes, completely dried under nitrogen, and diluted in 1 mL of methylene chloride. The extracted samples (1 μ L) were analyzed using an Agilent 7890A GC-FID equipped with an HP-5 capillary column (30 m × 0.32 mm × 0.25 μm; Agilent). The oven was held at 90°C for 2 min, increased at a rate of 4°C/min to 250°C, and then held this temperature for 18 min. The injector was operated in split mode (50:1) and held at 250°C. For the hydrocarbon analysis, ratios between the peak areas of selected saturate and aromatic hydrocarbons and the peak area of a squalene or *p*-terphenyl standard were calculated. Hydrocarbon loss was determined by comparing these area ratios of the oils from the inoculated columns relative to those from the uninoculated oil-containing columns. In addition, mass balance calculations for the bioconversion of hydrocarbons to methane were determined by estimating the amount (μ mol) of saturate and select aromatic hydrocarbons in each replicate and control samples based on calibration curves prepared with hexadecane (saturates) and *p*-terphenyl (aromatics), assuming a similar response factor for all saturates and aromatics. The amount of hydrocarbons consumed by the cultures was determined by subtracting the amounts in the inoculated, oil-amended column from those in the oil-amended, uninoculated control. Then, the expected amount of methane for that amount of hydrocarbon consumed was estimated for each hydrocarbon, based on stoichiometric reactions determined using the Symons and Buswell (1933) equation (Tables S1, S2).

## Results

### Methanogenic microbial activity and hydrocarbon metabolites

**Figure 1:**
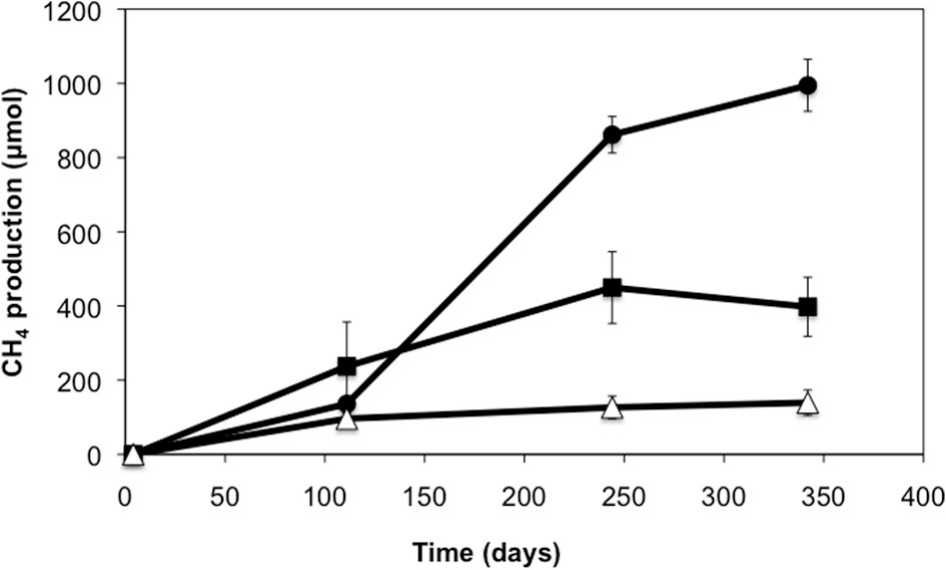
**Methane production in production water-derived incubations amended with light (circles) and heavy crude oil (squares) relative to oil-free controls (triangles)**. Error bars represent a standard deviation of the mean of triplicate incubations.

A crude oil-degrading enrichment culture was established from production waters of a heavy oil reservoir. After 28 weeks of incubation, enhanced levels of methane were observed in the methanogenic enrichments amended with light and heavy crude oil relative to oil-free controls (Figure 1). Enrichments amended with light oil produced higher methane (up to 860 μmol) than enrichments amended with heavy oil (up to 450 μmol). The average methane production rate for the light oil-amended incubations was 6.2 μmol CH_4_/g of oil/day, while that for the heavy oil amended incubations was 4.6 μmol CH_4_/g of oil/day. The pH of the enrichment culture was periodically checked and remained at pH 7, and acetate was not detected throughout the incubation period.

**Figure 2:**
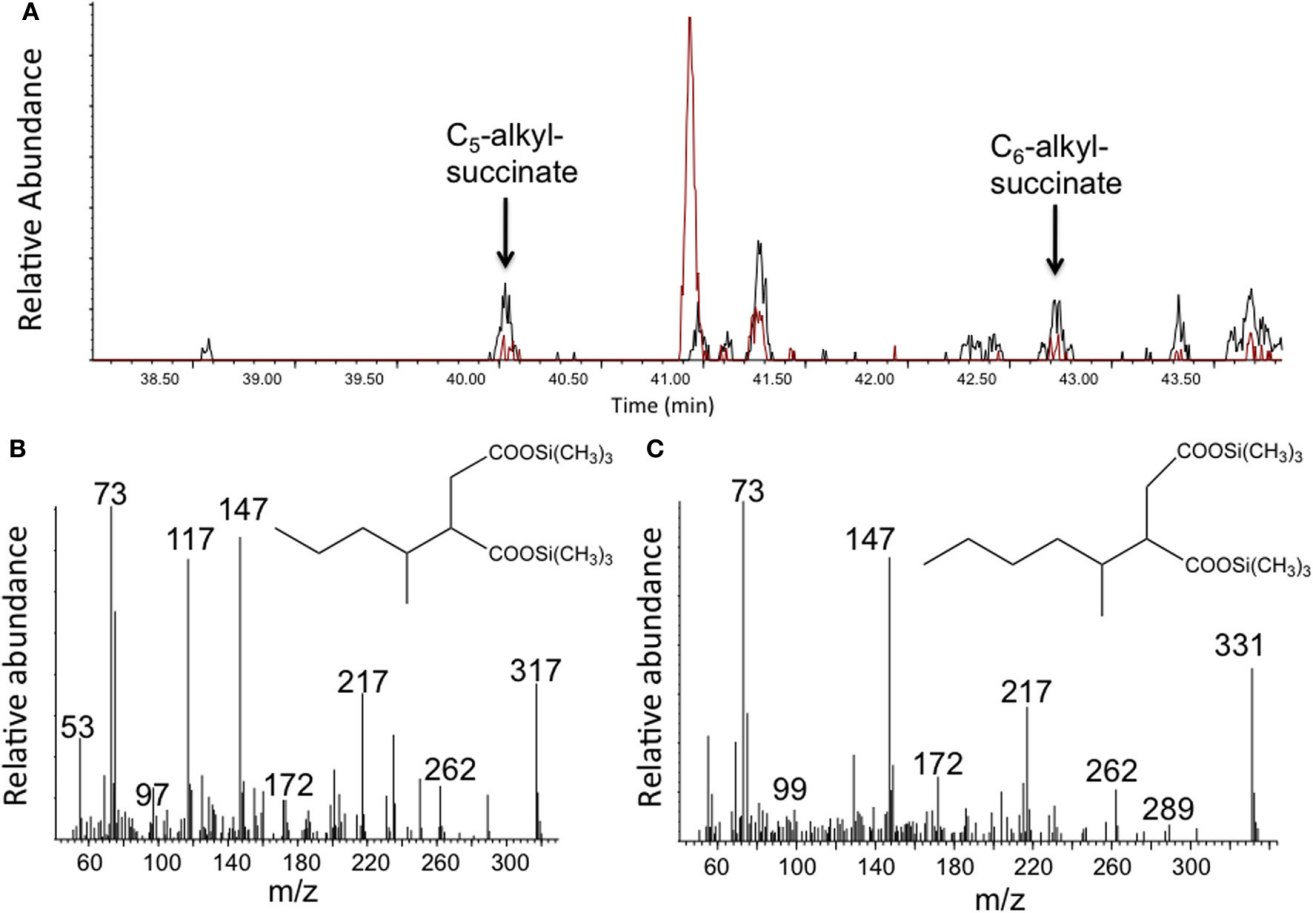
**Detection of putative alkylsuccinates in methanogenic crude oil-amended incubations. (A)** A portion of a GC total ion chromatogram showing larger peaks detected in oil-amended culture extracts (black) relative to oil-free controls (red) whose mass spectral profiles are indicative of **(B)** pentylsuccinate and **(C)** hexylsuccinate. Metabolites were detected as their trimethylsilylated derivatives.

To determine whether any hydrocarbon metabolites known to be formed under anaerobic conditions were present in the enrichment culture, the light oil-amended incubations and corresponding oil-free controls were subject to metabolite analysis after 302 days of incubation. Using GC-MS analysis of silylated organic extracts, we detected various peaks in the oil-amended culture that were either not present or that were observed in higher abundances than in the oil-free control incubations. For instance, MS analysis revealed the presence of benzoate (m/z 194, M^+^-15) and cyclohexane carboxylate (m/z 185, M^+^-15), known metabolites of anaerobic aromatic compound degradation, in the oil-amended culture only. Other metabolites had mass profiles corresponding to toluic acids (m/z 208, M^+^-15) and carboxybenzylaldehyde (m/z 222, M^+^-15), suggesting the degradation of monoaromatic hydrocarbons. We also detected peaks with MS fragment ions diagnostic of silylated fumarate addition metabolites of *n*-alkanes (e.g., alkylsuccinates), including methylsuccinate (m/z 276, 261, 217, 172, 147, 73), and presumed pentylsuccinate (m/z 317 (M^+^-15), 262, 217, 172, 147, 73) (Figure 2B), and hexylsuccinate (m/z 331 (M^+^-15), 262, 217, 172, 147, 73) (Figure 2C) (based on published MS profiles; Gieg and Suflita, 2002). Although these alkylsuccinate peaks were present at low abundance, they were clearly detected above the levels of those found corresponding oil-free controls (Figure 2A). Low levels of the metabolites were likely detected in the oil-free controls due to the presence of small amounts of oil transferred from the oil-amended enrichment in order to set up the oil-free controls. In accordance with these results, fumarate addition genes encoding for the enzymes involved in anaerobic degradation of alkanes (*assA*) and aromatic hydrocarbons (*bssA*) were also detected in the enrichment culture. We obtained a single fragment (771 bp) that showed homology (86%) to known benzyl succinate synthase subunit A sequences, and another fragment (501 bp) that also showed homology (92%) to known alkylsuccinate synthase gene sequences using primer sets from Callaghan et al. (2010) targeting *bssA* genes (1213F GACATGACCGAYGCCATYCT and 1987R TCRTCGTCRTTGCCCCAYTT) and *assA* genes (1432F CCNACCACNAAGCAYGG and 1936R TCRTCATTNCCCCAYTTNGG) (Figure S2).

### Residual oil-simulating columns

Following the establishment of an oil-degrading methanogenic enrichment culture, we evaluated its ability to utilize residual crude oil components entrained in a sandstone-packed column in order to more closely simulate a marginal oil field system. Methane production was periodically monitored in three replicate columns amended with the culture and residual crude oil, a control column containing the inoculum but no oil, and a control column containing oil but no inoculum. During the 315 days of incubation, enhanced levels of methane were observed in all the inoculated, oil-amended columns relative to the control columns. Moreover, one of the replicate columns produced the highest amount of methane (~160 μmol) after only 48 days of incubation. The methane production rate for the latter column was 5.84 μmol CH_4_/g of oil/day, slightly lower than the original enrichment culture (6.2 μmol CH_4_/g of oil/day). The other two replicate columns had lower methane production rates of 0.16 and 0.33 μmol CH_4_/g of oil/day. Up to 220 μmol CO_2_ were detected in the column with the highest methane production, while slightly lower amounts were detected in the other replicates (up to 129 and 200 μmol). In the parallel columns flushed with water or medium during preparation and inoculated with produced water, comparatively small amounts of CH_4_ were produced during the 315-days incubation period however differences were seen in the columns in which water or a minimal salts medium was used for the column preparation. Up to 4 μmol CH_4_ were measured in the produced water-inoculated columns prepared with water, while up to 15 μmol CH_4_ were measured in the columns prepared with medium. These results suggest that the presence of nutrients (e.g., that may have remained in the columns due to the flushing procedure) can lead to increased methane production. Due to the low amounts of methane measured in this produced-water inoculated column set, only the columns inoculated with the enrichment culture were further analyzed.

**Figure 3:**
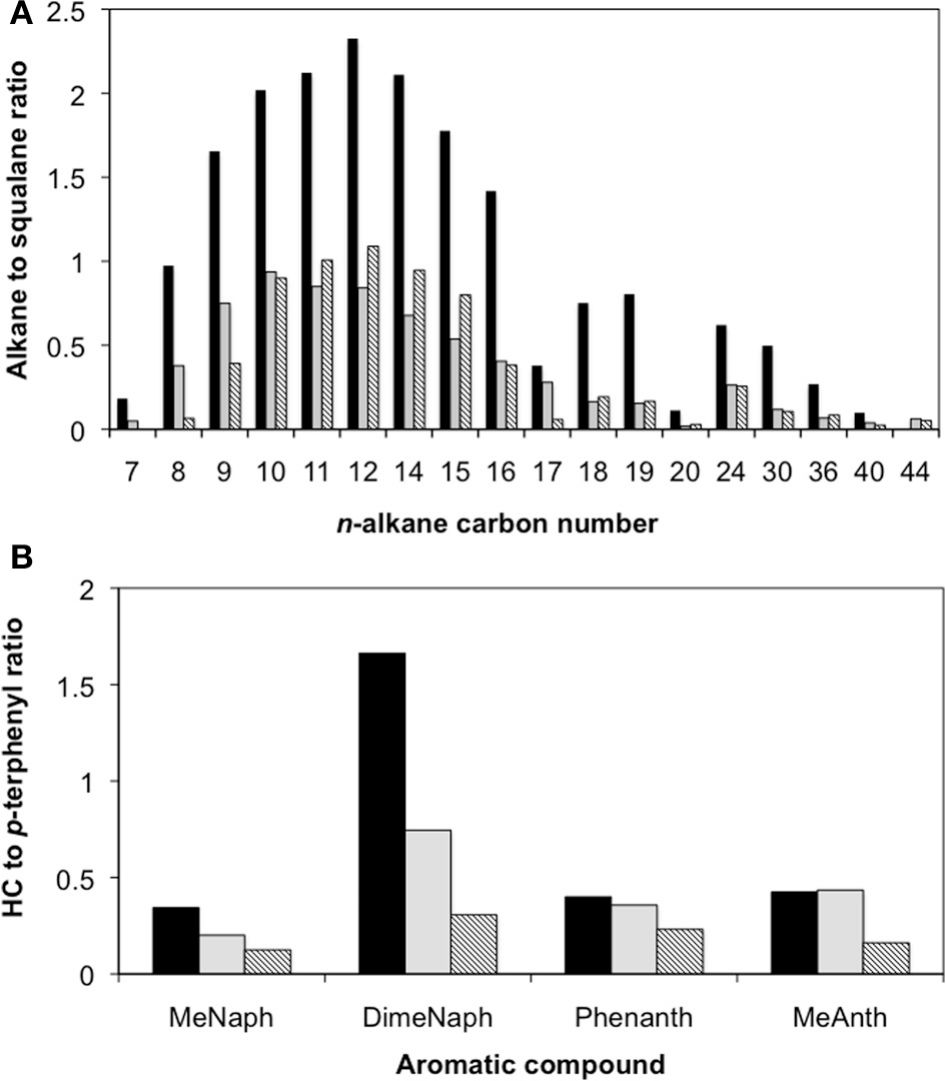
**Hydrocarbon loss as a function of the hydrocarbon (HC) to squalane peak area ratios for (A) *n*-alkanes ranging from C_7_ to C_44_, and (B) select polycyclic aromatic hydrocarbons after 315 days of incubation of a crude oil-degrading methanogenic enrichment in sandstone-packed residual oil-containing columns**. Striped bars represent the duplicate analyses from an inoculated column, while the black bars represent the uninoculated control. MeNaph, methylnaphthalene; DimeNaph, dimethylnaphthalene; Phenanth, Phenanthrene; and MeAnth, methylanthracene.

After 315 days of incubation, the enrichment culture-inoculated column producing the highest amount of methane as well as a control column containing oil only were sacrificed. Although pH determinations of the pore fluids were not made at the end of the experiment, the produced fluids (e.g., pore water) did not turn pink (e.g., indicating either acidic or high redox conditions) which was expected given that the medium used to prepare the columns was buffered at pH 7.1 with bicarbonate. Further, these observations align with the fact that the pH remained neutral during the course of hydrocarbon degradation in the planktonic enrichment culture. Acetate analysis showed that the aqueous fluids occupying the pore volume in the oil-containing column contained approximately 4 mM acetate, while no acetate was detected in the control column, suggesting oil metabolism in the live columns. The corresponding residual oils from each column were recovered and analyzed following separation into saturate and aromatic fractions. Between 20 and 80% of *n*-alkanes ranging from C_7_ to C_44_ were depleted in the replicate column relative to the uninoculated column (Figure 3A). Also, oil analysis showed that some identified PAHs (e.g., by matching retention times and mass spectra with authentic standards) such as methylnaphthalene, dimethylnaphthalene, phenanthrene, and methylanthracene were at least partially depleted in the inoculated replicate columns relative to inoculum-free control (Figure 3B). The two-ringed PAH were consumed to a greater extent than the three-ringed PAH analyzed (Figure 3B). According to mass balance calculations of the hydrocarbons that were quantified (e.g., the *n*-alkanes, the four known PAH compounds, and other 2- or 3-ringed aromatics), approximately 113 μmol CH_4_ were predicted to form in the column (Tables S1, S2). Experimentally, the most active column produced close to 160 μmol CH_4_ (Table S1). The difference in methane concentrations from predicted to actual amounts measured (~47 μmol) is likely due to the consumption of other hydrocarbons (such as branched alkanes or PAHs with >3 rings) that were not quantified in this study and were thus not taken into account in the mass balance calculations.

### Microbial community analyses

**Figure 4:**
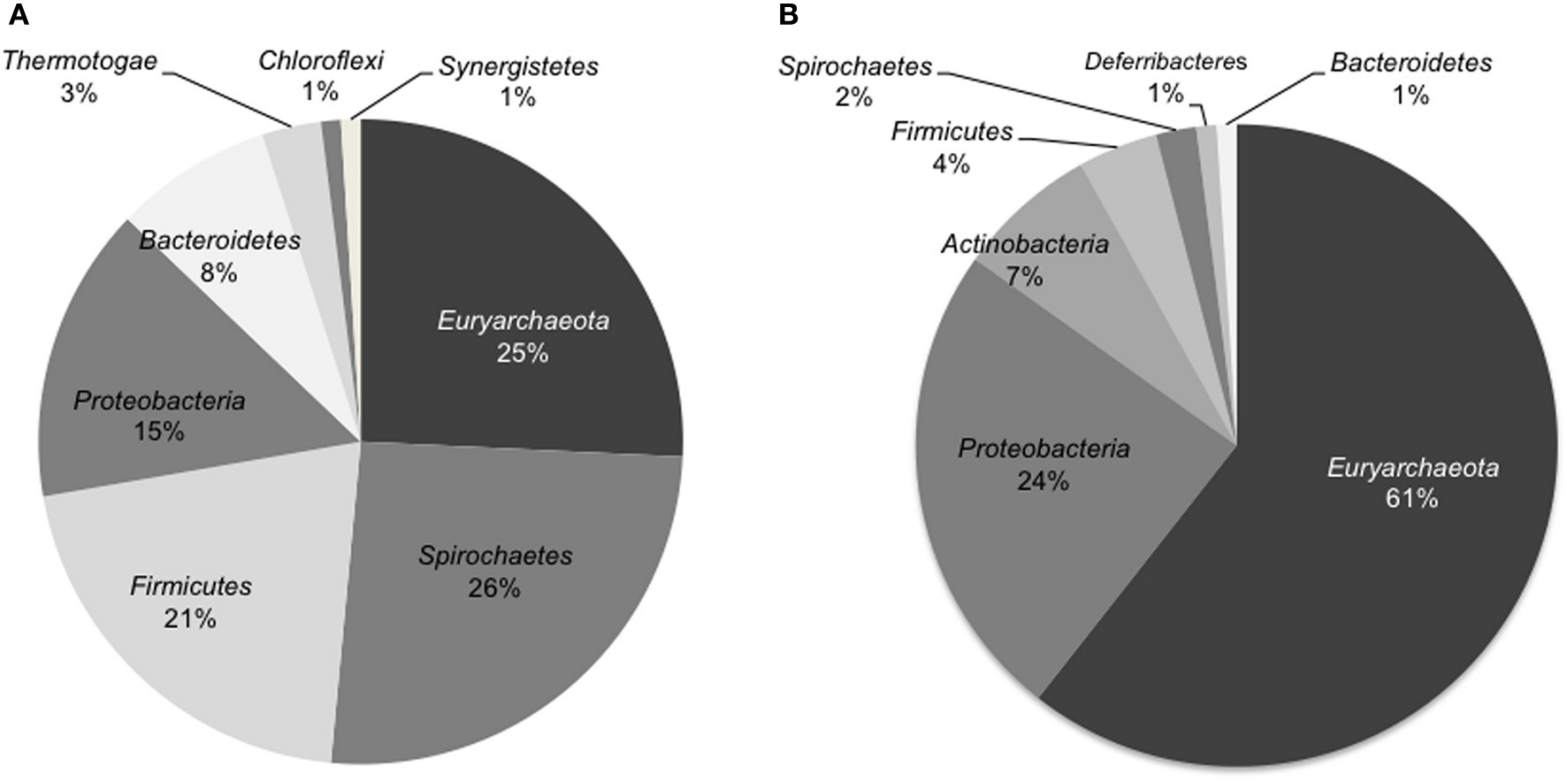
**Distribution of microbial sequence reads from pyrosequencing analysis identified at the phylum level of the 16S rRNA genes in the oil degrading methanogenic culture (A) and in the inoculated sand-packed column (B)**. Only abundances higher than 1% are shown.

The microbial community composition of the light-oil degrading enrichment before and after incubation in the sandstone-packed column experiment was assessed by pyrosequencing analysis of 16S rRNA genes. Overall, we observed a dramatic shift in the dominant microbial community members. First, the percentage of microbial reads from the *Archaea*, mainly methanogens, increased from 30% in the enrichment culture to 63% in the column system. At the phylum level, the community of the original enrichment culture was dominated by members of the phyla *Euryarchaeota, Spirochaetes, Firmicutes*, and *Proteobacteria* (Figure 4A). In contrast, the microbial community sampled from the residual oil column was dominated by members of *Euryarchaeota*, followed by members of *Proteobacteria*, and in less amount organisms from the phylum *Actinobacteria, Firmicutes*, and *Spirochaetes* (Figure 4B).

At the genus level, the microbial community of the enrichment culture was dominated by members of *Smithella* (27% total reads), followed by *Methanosaeta* (25% total reads) and *Methanoculleus* (23% total reads). Other microbial genera found in lower proportions (<5% total reads) in the enrichment included the methanogens *Methanocalculus* and *Methanolinea*, and members of the bacterial genera *Sedimentibacter, Desulfotomaculum, Desulfobacterium, Kosmotoga*, and *Thermanaerovibrio* (Table 1). In contrast, the composition of the community following incubation in the sandstone column for 315 days was dominated by *Methanobacterium* (56% of total reads at the genus level) and *Pseudomonas* (16%). Other microbial members comprising >1% of the total microbial reads identified at the genus level belonged to the genera *Methanocalculus*, *Eggerthella*, *Halomonas, Clostridium, Methanoculleus, Desulfovibrio*, and *Sedimentibacter* (Table 1).

**Table 1:** **Phylogenetic affiliations of the microbial reads identified at the genus level (at >0.1% abundance) by pyrosequencing of the 16S rRNA genes in the original oil-degrading methanogenic (planktonic) culture and after the inoculum was incubated in a sandstone-packed column**.

**Original enrichment culture**		**Sand-packed column culture**	
**Taxon (genus)**	**% of sequence reads**	**Taxon (genus)**	**% of sequence reads**
*Smithella*	27.57	*Methanobacterium*	55.90
*Methanosaeta*	25.48	*Pseudomonas*	16.33
*Methanoculleus*	22.95	*Methanocalculus*	4.91
*Methanocalculus*	5.52	*Eggerthella*	3.52
*Sedimentibacter*	5.23	*Halomonas*	3.20
*Desulfotomaculum*	3.27	*Methanoculleus*	2.88
*Desulfobacterium*	2.14	*Clostridium*	2.37
*Kosmotoga*	1.97	*Desulfovibrio*	1.28
*Thermanaerovibrio*	1.79	*Sedimentibacter*	1.14
*Petrotoga*	0.95	*Sphingomonas*	0.94
*Desulfuromonas*	0.53	*Methanosaeta*	0.84
*Syntrophorhabdus*	0.48	*Ralstonia*	0.69
*Desulfovibrio*	0.38	*Methylobacterium*	0.53
*Methanolinea*	0.25	*Geovibrio*	0.46
*Spirochaeta*	0.22	*Sediminibacterium*	0.39
*Tissierella*	0.20	*Methanomethylovorans*	0.37
*Leptolinea*	0.17	*Desulfuromonas*	0.34
*Fastidiosipila*	0.16	*Pelomonas*	0.30
*Anaerovorax*	0.12	*Tepidanaerobacter*	0.27
Others	0.64	*Burkholderia*	0.25
		*Bacillus*	0.23
		*Brevundimonas*	0.21
		*Spirochaeta*	0.21
		*Microbacterium*	0.18
		*Alicyclobacillus*	0.18
		*Methanofollis*	0.16
		*Thauera*	0.16
		*Koelreuteria*	0.16
		*Rhodococcus*	0.14
		*Thiobacillus*	0.11
		*Bradyrhizobium*	0.11
		Others	1.26

## Discussion

In this work, a methanogenic consortium enriched from production waters of a low-temperature oil reservoir was found to be capable of utilizing hydrocarbon components in crude oil. Although the demonstration of hydrocarbon-degrading oilfield-derived consortia remained elusive for many years, this finding adds to the handful of reports that now exist showing that microbes enriched from oilfield fluids can utilize hydrocarbons under methanogenic conditions (Gieg et al., 2010; Mbadinga et al., 2011; Zhou et al., 2012; Cheng et al., 2013).

The detection of putative anaerobic hydrocarbon metabolites at higher levels in the oil-amended than in the oil-free columns provides further evidence that the consortium metabolizes crude oil components. The detection of alkylsuccinates (Figure 2), along with alkyl- and benzylsuccinate synthase genes, suggests that addition to fumarate is at least one mechanism being used by a member(s) of the enrichment culture to activate hydrocarbons. Fumarate addition to *n*-alkanes has been proposed to occur in methanogenic microbial enrichments (Callaghan et al., 2010; Zhou et al., 2012) and oil-contaminated environments (Callaghan et al., 2010) where fumarate addition genes (*assA*) have been detected. However, detecting the products in laboratory cultures has proven difficult (e.g., Gieg et al., 2010; Zhou et al., 2012). Using a combined approach of metabolite analysis and qPCR analysis of the *assA* gene in a different methanogenic crude oil-degrading enrichment culture, Aitken et al. (2013) did not detect alkylsuccinates nor increases in copy numbers of the *assA* gene at significant levels above controls even though *n*-alkanes were metabolized over time. These results suggested that an alternate mechanism for activation of alkanes was occurring by members of this methanogenic consortium. In contrast, Tan et al. (2013) were able to detect fumarate addition metabolites from branched alkanes (but not from *n*-alkanes) in a short chain alkane-degrading microbial culture enriched from oil sands tailings ponds, which corresponded to the identification of *assA* in the metagenome of the consortium. These differing results demonstrate that the activation of hydrocarbons under methanogenic conditions remains uncertain, and that multiple (unidentified) mechanisms may occur, likely depending on the microbial/genetic composition of the consortium under study. In the present culture, alkylsuccinates were only assessed at one time point, thus additional work to determine whether these form transiently during methanogenic crude oil degradation is a goal of future work.

The microbial characterization of the oil degrading enrichment culture described here revealed that bacterial members of the genus *Smithella* dominated (Table 1). *Smithella* is a syntrophic member of the *Deltaproteobacteria* shown to be prevalent in several methanogenic oil-degrading cultures or environments (Gray et al., 2010). Recently, Gray et al. (2011) observed increased numbers of 16S rRNA genes from members of the genera *Smithella* and *Syntrophus* during methanogenic oil degradation. In a separate culture, a member of the *Syntrophaceae* closely related to *Smithella propionicus* was identified by DNA-SIP analysis as a key player in the methanogenic degradation of hexadecane (Cheng et al., 2013). Other organisms detected in abundance in our enrichment culture were *Sedimentibacter* and *Desulfotomaculum* species. Members of these genera have been detected in other hydrocarbon-degrading enrichments, and have been proposed to act as either primary or secondary syntrophs in such consortia (Kleinsteuber et al., 2012). In addition, sequencing analysis revealed that acetate- and H_2_-utilizing methanogens in the enrichment culture are found in similar proportions in the enrichment culture. The fact that acetate was not detected during incubations of the enrichment culture with crude oil suggested acetate was effectively consumed by the acetotrophic methanogens. To date, there is no clear consensus regarding the predominant route (via acetotrophic or hydrogenotrophic methanogenesis) involved in methanogenic hydrocarbon biodegradation since both mechanisms have been observed in hydrocarbon studies (Gieg et al., 2008; Jones et al., 2008). However, further experiments are required to assess the specific roles that these abundant organisms play in the anaerobic degradation of crude oil components.

In an effort to determine how hydrocarbon methanogenesis would proceed in a system more closely resembling a marginal oilfield, we inoculated the consortium into sandstone packed columns containing residual oil. Although the columns were not prepared under pressurized conditions (e.g., that would more truly simulate an actual oilfield), they were used as a “proof-of-concept” assessment of whether hydrocarbon methanogenesis would occur in a system characterized by residual oil-laden porous rock. From the column experiments, we were able to measure the evolution of methane at rates of up to ~6 μmol CH_4_/g of oil/day, and after a “shut-in” period, more than 50% hydrocarbons were biodegraded, presumably to methane, in inoculated columns relative to uninoculated controls (Figure 3). Thus, the methanogenic consortium was able to utilize crude oil components both as a planktonic culture, and in a sessile environment simulated using sandstone-packed columns. Notably, however, the microbial community composition of the enrichment shifted substantially when grown planktonically in liquid medium vs. growth on a solid support (Figure 4, Table 1). First, the relative abundance of methanogens increased, especially *Methanobacterium* (a H_2_-user), in the sessile community relative to the original planktonic enrichment culture (Figure 4, Table 1). *Methanocalculus* (H_2_-user) remained at a similar abundance (~5% of total reads at the genus level), while the relative abundances of *Methanoculleus* (H_2_-user) and *Methanosaeta* (acetate-user) decreased substantially. These data align with the acetate measurements conducted during this study. In the planktonic enrichment culture, both acetotrophic and hydrogenotrophic methanogens were almost equally abundant (as deduced by pyrotag sequencing) and no acetate was detected during the incubation suggesting effective acetate consumption by the acetotrophic methanogens (e.g., *Methanosaeta* comprised 25% of reads at the genus level). At the end of the column experiment when acetate could be measured upon opening the columns, approximately 4 mM acetate had accumulated in the pore water fluids, suggesting less effective acetate removal by the sessile community. Indeed, *Methanosaeta* comprised <1% of the sequence reads at the genus level (Table 1) at the end of the column experiment. It is currently not known why the sessile, column incubations favored the predominance of H_2_-using methanogens such as *Methanobacterium*, as both H_2_- and acetate-using methanogens have been noted to comprise biofilms used for a variety of industrial applications (Calderón et al., 2013). A second striking shift observed in the planktonic vs. sessile experiment is that bacterial members of the genus *Pseudomonas* dominated the community of the sand-packed column, whereas the most predominant bacterial members in the original enrichment were related to *Smithella*. This was a surprising find because *Pseudomonas* species are often described as hydrocarbon-degrading aerobes, while the columns incubations were strictly anoxic. However, these organisms are facultative and have been associated with oil biodegradation under some anoxic conditions (Grossi et al., 2008). Further, *Pseudomonas* spp. have been detected in samples collected from anoxic oil reservoirs (Li et al., 2012; Zhang et al., 2012a; Meslé et al., 2013). The proliferation of *Pseudomonas* sp. in the sandstone column environment may be related to the known ability of these organisms to form biofilms (e.g., Klausen et al., 2006; Li et al., 2009) thus giving them a competitive advantage in sessile environments. *Halomonas* sp., also enriched in the sandstone columns (Table 1), are also known biofilm and exopolysaccharide-forming organisms that may allow for the solubilization of hydrocarbons attached to rock (Llamas et al., 2006; Qurashi and Sabri, 2012; Gutierrez et al., 2013). Members of this genus have also been found to be associated with oil reservoirs and the deep biosphere (Mnif et al., 2009; Dong et al., 2013). The enrichment of *Eggerthella* in the sandstone incubations is unclear, given that this organism is a known gut-associated organism, although clones related to this genus (e.g., in the *Actinobacteria*) were identified in a different methanogenic crude oil-degrading consortium (Gieg et al., 2008). Other bacteria that were enhanced in the column were members of the genera *Clostridium* and *Desulfovibrio*. These bacteria and other members from the *Firmicutes* and *Deltaproteobacteria* groups have been associated with anaerobic oil biodegradation (Gray et al., 2010). Given the relatively high abundance of *Pseudomonas* in the sandstone-packed column experiment, we hypothesize that these bacteria were at least partially responsible for the anaerobic biodegradation of crude oil components, potentially acting as syntrophic organisms in conjunction with methanogens. While such a hypothesis remains to be tested, it should be noted that pyrotag sequencing of several produced water samples from the oilfield from which the enrichment was derived revealed the presence of *Pseudomonas* sp. and other nitrate-reducers along with methanogens (Agrawal et al., 2012) thus interactions between these kinds of microbes may be occurring in some oilfield environments. Interestingly, the coexistence between these two groups of microorganisms in bioreactor systems have been previously noted (Percheron et al., 1999). Further, although it seems unusual that taxa with known aerobic metabolism would be detected in anoxic incubations, a recent genomic survey of petroliferous deposits revealed the presence of many aerobic taxa in known anoxic environments such as deep coal seams and oil sands deposits (An et al., 2013). Collectively, such findings clearly warrant further investigation to deduce the ecological role of such taxa in methanogenic environments such as oil reservoirs. Although cell growth was not monitored in the columns (or in the original planktonic enrichment), future work quantifying the key organisms identified in this study using an approach such as qPCR will help address their growth and role in response to crude oil substrates under both planktonic and sessile conditions.

Overall, the results of the present study demonstrate that the methanogenic microbial consortium enriched from oilfield production waters can convert hydrocarbons to methane either planktonically or as a sessile community. Further, microbial community sequencing analysis showed the dominance of different microbial taxa in the different incubation systems, suggesting that different kinds of microbes function to degrade hydrocarbons associated with solid or liquid phases in petroleum reservoirs.

### Conflict of interest statement

The authors declare that the research was conducted in the absence of any commercial or financial relationships that could be construed as a potential conflict of interest.
