# Supplementary material for: Conversion of crude oil to methane by a microbial consortium enriched from oil reservoir production waters
Source: Front Microbiol. 2014 May 5;5:197. doi: 10.3389/fmicb.2014.00197 (PMC4017130; doi:10.3389/fmicb.2014.00197)
Supplement: Supplementary file 1 [file DataSheet1.PDF]

Supplementary Information

Berdugo-Clavijo and Gieg,
Conversion of Crude Oil to Methane by a Microbial Consortium Enriched From Oil Reservoir Production Waters

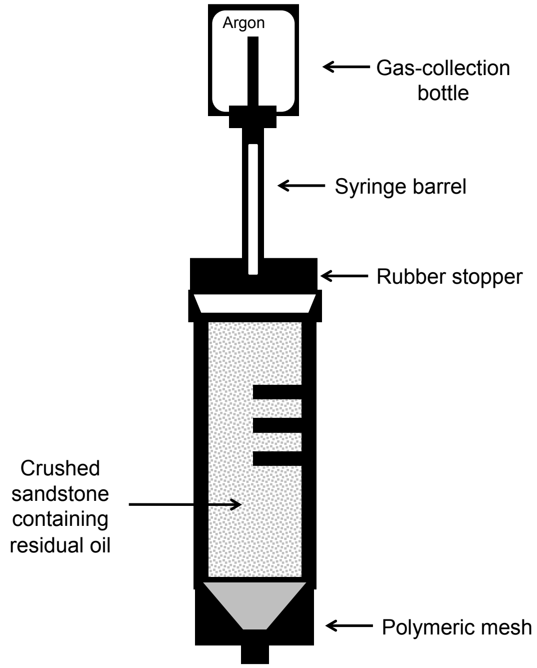

Figure S1. Diagram of a sandstone-packed column simulating a residual oil containing system.

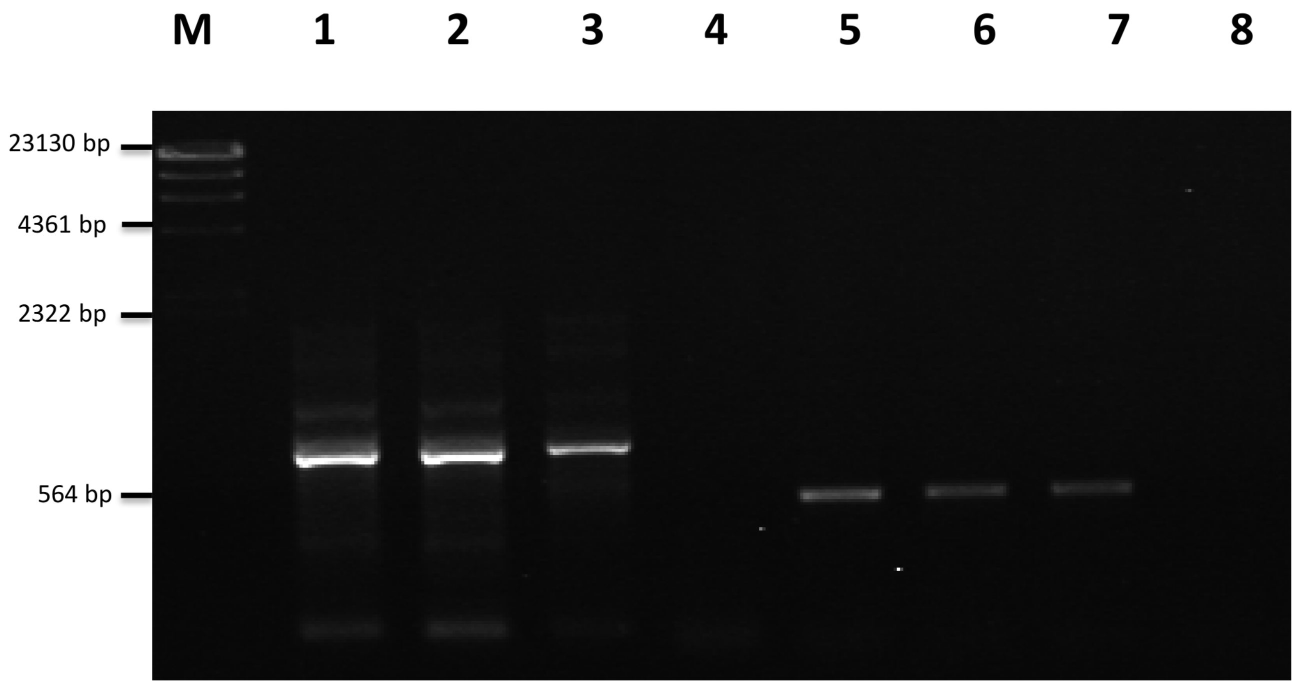

Figure S2. Agarose gel showing amplified bands of expected sizes from alkyl- or benzyl-succinate synthase gene analysis of the crude oil degrading enrichment culture. M: DNA marker, lanes 1-3: replicate samples with primer *bssA* set #2 (793 bp), lane 4: negative control with primer set #2, lanes 5-7: replicate samples with primer *assA* set #7 (523 bp), and lane 8: negative control with primer set #7. Primer sets used were from Callaghan et al., 2010, *Environ. Sci. Technol*. 44**,** 7287-7294.

Table S1. Amounts (µmol) of *n*-alkanes detected in oil-containing uninoculated and inoculated sandstone-packed columns and expected amounts (µmol) of methane based on predicted stoichiometric reactions and actual losses of hydrocarbons. These data were generated by accurately quantifying the listed *n*-alkanes.

	µmol of *n*-alkane			µmol of *n*-alkane consumed		µmol CH_4 per one µmol of *n*-alkanes	Expected CH_4 based on alkane consumed (µmol)	
***n*-alkane C#**	**Un-inoculated Control**	**Replicate 1**	**Replicate 2**	**Replicate 1**	**Replicate 2**		**Replicate 1**	**Replicate 2**
7	0.51	0.41	0.00	0.09	0.51	5.5	0.51	2.78
8	1.05	0.64	0.42	0.41	0.63	6.25	2.58	3.93
9	1.52	0.90	0.65	0.63	0.87	7	4.38	6.12
10	1.78	1.03	1.00	0.75	0.77	7.75	5.80	6.00
11	1.85	0.97	1.08	0.88	0.77	8.5	7.48	6.56
12	1.99	0.96	1.13	1.03	0.86	9.25	9.50	7.91
14	1.84	0.85	1.03	0.99	0.80	10.75	10.65	8.65
15	1.61	0.75	0.93	0.86	0.68	11.5	9.86	7.78
16	1.36	0.66	0.64	0.70	0.72	12.25	8.59	8.79
17	0.64	0.57	0.42	0.07	0.22	13	0.90	2.89
18	0.90	0.49	0.51	0.41	0.39	13.75	5.59	5.32
19	0.94	0.49	0.49	0.45	0.44	14.5	6.52	6.40
20	0.46	0.39	0.40	0.07	0.06	15.25	0.99	0.88
24	0.81	0.56	0.56	0.25	0.25	18.25	4.51	4.61
30	0.72	0.46	0.45	0.26	0.27	22.75	5.97	6.17
36	0.56	0.43	0.44	0.14	0.13	27.25	3.80	3.47
40	0.45	0.41	0.39	0.04	0.05	30.25	1.26	1.59
44	0.44	0.42	0.41	0.02	0.02	33.25	0.55	0.79
	**Total alkane consumed (µmol)**			8.03	8.44	**Expected CH_4 (µmol)**	89.44	90.64

Table S2. Amounts (μmol) of aromatic compounds detected in uninoculated and inoculated sandstone-packed columns and expected amounts (μmol) of methane based on predicted stoichiometric reactions and actual losses of hydrocarbons. These data were generated by accurately quantifying the listed aromatic hydrocarbons, and then assuming that nearby peaks on the GC chromatograms were various isomers of these 2- or 3-ringed PAHs, thus these calculations are estimates.

Sum of aromatic analogs	μmol of aromatics			μmol of aromatics consumed		μmol CH_4 per one μmol of aromatic	Expected CH_4 based on aromatic consumed (μmol)	
	Control	Replicate 1	Replicate2	Replicate 1	Replicate 2		Replicate 1	Replicate 2
MeNaph	0.071	0.062	0.067	0.009	0.004	6.75	0.061	0.028
DimeNaph	3.217	2.037	4.427	1.180	0.085	7.5	8.850	0.649
Phenanth	1.673	1.129	1.876	0.544	0.060	8.25	4.623	0.856
MePhenanth	4.547	2.721	3.961	1.826	1.502	9	18.095	12.540
	**Total μmol of aromatics consumed**			3.559	1.651	**Expected CH_4 (μmol)**	31.629	14.074
